# Determinants of egg consumption by infants and young children in Ethiopia

**DOI:** 10.1017/S1368980022001112

**Published:** 2022-11

**Authors:** Bezawit E Kase, Edward A Frongillo, Sejla Isanovic, Wendy Gonzalez, Hana Yemane Wodajo, Eric W Djimeu

**Affiliations:** 1 Department of Epidemiology and Biostatistics, University of South Carolina, 915 Greene Street, Columbia, SC 29208, USA; 2 Department of Health Promotion, Education, and Behavior, University of South Carolina, Columbia, SC, USA; 3 Global Alliance for Improved Nutrition, Geneva, Switzerland; 4 Global Alliance for Improved Nutrition (GAIN) Ethiopia Country Office, Addis Ababa, Ethiopia; 5 Global Alliance for Improved Nutrition, Washington, DC, USA

**Keywords:** Egg, Complementary feeding, Child nutrition, Infant nutrition, Children, Infants, Nutrition

## Abstract

**Objective:**

To identify determinants of egg consumption in infants and young children aged 6–23·9 months in Ethiopia.

**Design and setting:**

Data used were from the cross-sectional baseline survey of an egg campaign in Ethiopia implemented by the Global Alliance for Improved Nutrition.

**Participants:**

Children aged 6–23·9 months (*n* 453) were sampled. Data on socio-demographic characteristics, economic resources, caregiver’s behaviour, child health and feeding practices, and egg consumption in the last 7 d were collected using interviewer-administered questionnaires. Multivariable ordinal logistic regression was used to examine the association between explanatory variables and egg consumption in the last 7 d.

**Results:**

About half of children (53·4 %) did not consume eggs in the last 7 d. The odds of children consuming eggs were 4·33 (*P* < 0·002) times higher when their caregivers had some college education compared with no education. Wealth was positively (OR, 1·13, *P* = 0·029) and household food insecurity was negatively (OR, 0·96, *P* = 0·117) associated with child egg consumption. Purchasing eggs (OR, 9·73, *P* < 0·001) and caregiver’s positive behavioural determinants (OR, 1·37, *P* = 0·005) were associated with child egg consumption. The associations of socio-demographic characteristics and economic resources with egg consumption provide evidence of partial mediation through caregiver behaviour and child health.

**Conclusions:**

About half of children aged 6–23·9 months consumed eggs. Availability of eggs in households, mainly through purchase, was strongly associated with egg consumption. Education of caregivers and household heads and economic resources were associated with egg consumption and may operate through caregiver behaviour.

Adequate nutrition for infants and young children is critical for healthy growth and development. Inadequate complementary feeding can lead to early nutritional deficiencies that impact a child’s ability to reach their full potential in adulthood^(1)^. Animal-source foods are an important part of complementary feeding because these foods can provide critical nutrients^(1)^.

Eggs are a nutritionally dense, animal-source food that have the potential to improve early nutrition^(2,3)^. Eggs are a source of protein, minerals such as Se and K, vitamins such as riboflavin and vitamin B_12_, and essential fatty acids, particularly DHA, all of which are beneficial to child growth and development^(4)^. Introducing eggs during complementary feeding in areas where stunting is prevalent has been shown to promote growth, amongst other nutritional benefits^(5)^. The beneficial properties of eggs extend beyond their nutritional content. In comparison with other nutrient-dense animal-source foods, eggs can be easily stored and prepared^(6)^. These beneficial properties have spurred focus on promoting egg consumption through interventions^(3,5,7,8)^. Developing effective interventions requires understanding the economic, physical, cultural and behavioural determinants of egg consumption among children.

The availability of chickens, ample agricultural land for egg production, access to markets selling eggs and food security are possible influences on egg consumption^(9–12)^. Cost of egg production and purchase may inhibit egg consumption^(3,12–14)^. Among women and young children in rural Zambia, cost was identified as one of the primary obstacles to sustain egg consumption. Although many women were favourable to children consuming eggs, the high price of eggs in rural Zambia resulted in many households opting to sell eggs and chickens rather than keeping them for consumption purposes^(13)^.

Culturally based norms can shape the perception of egg quality, thus affecting the frequency of egg consumption^(12,15)^. Caregivers’ perspectives are often related to credibility, personal experience and culturally based norms and values^(12)^. Religious beliefs and practices can affect egg consumption^(16)^. In the Ethiopian Orthodox Christian religion, extensive fasting (180 d per year on average) is practiced in which animal-source foods are not consumed and are less available in markets^(17)^. In a previous study in Ethiopia, only a quarter of children 6–23 months of age consumed animal-source foods and one-third of households had animal-source foods available in the house during fasting period^(16)^.

Hindrances for egg consumption in children may be contextual, which can be shown by previous studies conducted in different countries. Recent qualitative and mixed-methods studies conducted in different countries have garnered information related to child egg consumption, reporting associations between chicken ownership and higher egg consumption by women and children in Burkina Faso, Tanzania, Senegal and Cote d’Ivoire^(14)^, limited availability of chickens and eggs and low frequency of egg consumption by mothers and their children in Tanzania^(11)^, and women’s perceptions of food practices, egg quality, forms of preparation and cost, and frequency of household egg consumption in Ecuador^(12)^. Nevertheless, substantial gaps in knowledge exist concerning the determinants of variation of egg consumption in the cultural and economic context of Ethiopia. Except for one study that investigated consumption of animal-source foods^(16)^, no quantitative study has comprehensively examined the possible modifiable and non-modifiable determinants of egg consumption among infants and young children in Ethiopia. This study aimed to identify how socio-demographic characteristics, economic resources, caregiver behaviour and child health explain variation in egg consumption in infants and young children aged 6–23·9 months in Ethiopia.

## Methods

### Data source and study population

This cross-sectional study used data from the baseline survey collected from October to November 2019 as a part of the planned evaluation of an egg promotion campaign in Ethiopia implemented by the Global Alliance for Improved Nutrition. The campaign was intended to use behavioural change interventions to improve dietary quality of under-five children in Ethiopia by promoting consumption of eggs. The behavioural change intervention focused on increasing demand for nutritious foods, mainly eggs, to be consumed by children 6–59 months. The campaign encourages parents to feed children at least two eggs per week. The demand creation activities were implemented through Above the Line and Below the Line campaigns. Above the Line activities were developed to reach and inform the public through fun and informative engagement. The Above the Line activities included radio messages, bajaj branding, billboard mounting and digital promotion containing messages that promote the benefit of egg consumption. Below the Line activities were designed to engage consumers personally and prompt consumers to purchase eggs in the market. The Below the Line activities included market activation, road shows, sensitisation workshops to key community groups, door to door promotion and mobile promotion in collaboration with creative agencies and health extension workers.

The baseline survey and the campaign were conducted in Southern Nations, Nationalities, Peoples’ Region; Oromia; Tigray; and Amhara regions of Ethiopia. These regions collectively account for the majority of the Ethiopian population from different corners of the country (different geography and weather conditions). Multi-stage sampling was used to acquire a representative sample of children less than 5 years of age and households with at least one eligible child living in urban/semi-urban areas in Ethiopia. From two of the four regions (i.e. Southern Nations, Nationalities, Peoples’ Region and Oromia), four towns per region were selected, and from the other two regions (i.e. Tigray and Amhara), two towns per region were selected. At stage one of the sampling, kebeles (the smallest administrative unit in Ethiopia and equivalent to neighbourhood associations) within towns were selected using a lottery method for towns with many kebeles (more than the needed number of kebeles within a town); otherwise, all kebeles within the towns were included. The number of kebeles per town in the sample was determined based on probability proportionate to size, and a total of 50 kebeles were included. At stage two, sampling frames listing all households with at least one under-five child per each kebele were produced, and systematic random sampling was used to select the required number of households. Equal number of households were selected for each kebele. A total of 1175 households were sampled. For the current study, the study population was infants and young children aged 6–23·9 months (*n* 453). This age group was selected because it is important to examine how eggs are incorporated in the complementary feeding period.

Data were collected using an interviewer-administered structured questionnaire (see online Supplementary Material). Mothers or primary caregivers of participating children provided a written informed consent and completed the interview during a household visit. Ethical clearance was obtained from Ethiopian Public Health Institute (EHI-IRB-184-2019).

### Conceptual model

Based on the Theory of Planned Behavior and Social Cognitive Theory^(18)^, a conceptual model of determinants of egg consumption in infants and young children was created using variables available from the baseline survey. We posited that low egg consumption in infants and young children results from low educational level of caregivers and head of households, low economic status (indicated by wealth and occupation), high household food insecurity and low access to eggs. These socio-demographic characteristics and economic resources make up the distal layer of determinants that influence behaviour^(18)^. Socio-demographic characteristics and economic resources may determine egg consumption mediated through caregiver’s behaviour and child health. The caregiver’s attitude towards feeding eggs to children, intent to feed eggs, self-efficacy to feed eggs, social norms, decision making and household availability of eggs were posited to be proximate determinants that drive behaviour leading to egg consumption^(18)^. We also expected higher egg consumption in infants and young children who consumed a higher diversity of other foods and had better health and appetite.

### Measures

#### Egg consumption in children 6–23·9 months of age

Caregivers were asked to report if their child consumed eggs in the last 7 d. The caregivers who affirmed their child consumed eggs were then asked a follow-up question requesting the number of eggs the child consumed during those 7 d. These two variables were combined to create the primary outcome, an ordinal scale. Caregivers who did not feed eggs to their child in the last 7 d were asked to report their reason for not feeding eggs. Caregivers also reported if a child consumed eggs in the last 24 h, which we used as a binary (yes/no) secondary outcome to confirm our findings in a sensitivity analysis.

#### Socio-demographic and economic resource variables

In the baseline survey, the following characteristics were reported for the caregiver: sex, age (years), marital status, religion, education status and occupation. For the head of household: sex, age and education were reported. The child socio-demographic variables were sex and age.

An asset-based wealth index was constructed in accordance with the Ethiopia Demographic and Health Survey, 2016^(19)^. A principal component analysis of thirty-eight variables was used to construct the wealth index. In predominantly poor populations, higher scores on the wealth index correspond to greater wealth.

Household food security was measured using the Household Food Insecurity Access Scale^(20)^. A sum score was computed (range 0–27) with higher scores representing greater food insecurity.

Caregivers reported distance to markets (min) and whether eggs are available (yes/no) in the markets they frequently visit. To assess the availability of eggs in the households, caregivers were asked if eggs were produced in the household (yes/no) and if eggs were purchased (yes/no) in the last month.

#### Caregiver behaviour

Caregiver behaviour was assessed based on the constructs of caregiver’s attitude towards feeding eggs to children, intent to feed eggs, self-efficacy to feed eggs and social norms, drawing on the Theory of Planned Behavior and Social Cognitive Theory. The study team included in the baseline questionnaire items that reflected each of these constructs.

Caregivers’ positive and negative attitudes towards feeding eggs to children were measured. Positive attitudes were measured using eleven items with responses on a Likert scale. The responses for these items (each with range 1–5) were used to compute a mean score, with higher scores corresponding to caregivers’ favourable attitudes towards feeding eggs to children. Similarly, negative attitudes towards feeding eggs to children were assessed using ten items (each with range 1–5). The mean score of these items was obtained with higher score corresponding to a disapproval of feeding eggs to children. Caregiver’s intention to feed eggs was assessed using a single item on a Likert scale (range 1–5). Caregiver’s self-efficacy to feed eggs to the child was assessed by two items with responses on a Likert scale (range 1–5), and a mean score of these items was computed. Social norms that addressed feeding eggs to children were assessed by four items with responses on a Likert scale (range 1–5), and the mean of these items was computed. Cronbach’s *α* was computed for each of the caregiver behaviour variables using the *proc corr* procedure with option *Alpha* in SAS. Cronbach’s *α* for positive attitude (0·79), negative attitude (0·82), self-efficacy (0·77) and social norms (0·70) indicated good internal consistency. To complement Cronbach’s *α* as an estimate of internal consistency, the extent to which items appeared to be eliciting the same underlying construct^(21)^ was evaluated by experts in the field prior to data collection to ensure construct validity following existing guidance on developing tools that measure components of Theory of Planned Behavior and Social Cognitive Theory.^(22)^

Caregivers who reported purchasing eggs in the past month were asked to report the maximum amount of money (Ethiopian Birr, EBT) they were willing to spend on purchasing eggs in the past month. Higher amounts of money reported by caregivers indicated a greater willingness to purchase eggs. Caregivers were asked if they recognise receiving messages promoting feeding eggs to children (eggs make children strong and active or bright and sharp) from one of the following sources: radio or television, healthcare workers or someone in the community.

Children’s food diversity was assessed using a 24-h recall of consumption of seven food groups: grains and roots, legumes, dairy products, flesh foods, eggs, fruits and vegetables rich in vitamin A and other fruits and vegetables. With the exclusion of eggs, the number of food groups a child consumed, ranging from 0 to 6, was used in the analysis.

### Data analyses

Descriptive statistics were obtained using means with standard deviations and percentages. Bivariate associations between potential determinants and egg consumption were performed using binary ordinal logistic regression. The OR from the exponentiated coefficients quantify the odds of consuming more eggs compared with fewer eggs in relation to the explanatory variables.

Preliminary analysis showed that a caregiver’s positive attitudes towards feeding eggs to children, self-efficacy, social norms and intention to feeds eggs was highly associated with each other as expected from the Theory of Planned Behavior and Social Cognitive Theory^(18)^. Therefore, a new variable, positive behavioural determinants, was constructed from these four variables using factor analysis, assuming one factor to obtain a factor score. Similarly, the ranking of a child’s health and appetite by caregivers were highly associated, and therefore the sum of the two rankings was computed.

**Fig. 1:**
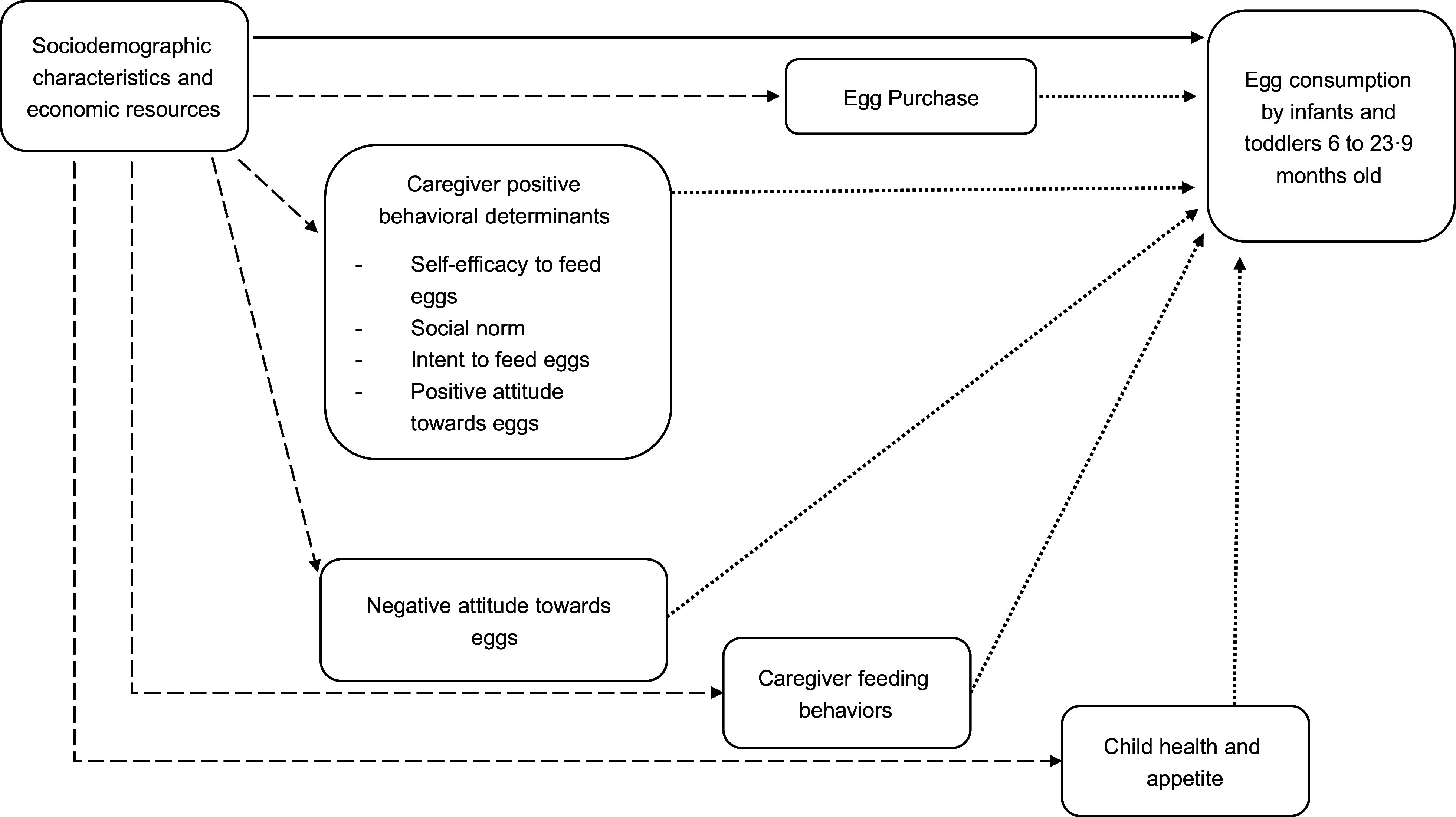
Analytic framework. Model A included relationships indicated by solid lines. Model B included relationships indicated by dotted lines. Model C included relationships indicated by both solid and dotted lines. Mediation paths that were implied by model C but not directly examined are indicated by dashed lines

Multivariable ordinal logistic regression was used to examine the association between explanatory variables and the ordinal egg consumption. Three models were compared (Fig. 1). Model A included only the socio-demographic characteristics and economic resources. Model B included only caregiver behaviour and child health. Model C included both sets of variables. Comparison of models A and C assessed mediation of socio-demographic characteristics and economic resources through caregiver behaviour and child health using the difference method^(23)^. We examined statistical interactions between caregiver’s education and caregiver’s attitudes, caregiver’s education and purchasing eggs, wealth and caregiver’s attitudes, wealth and purchasing eggs, household food insecurity and caregiver’s attitudes, and household food insecurity and purchasing eggs. Models A–C also were run stratifying children into two age groups, 6–14·9 and 15–23·9 months; results from the two age groups were similar and not reported. All analyses were performed using SAS version 9.4 and Stata version 15, accounting for clustering of kebeles within towns using a sandwich estimator. OR and *P*-values are reported to show the strength and confidence in the associations, and C-statistics are reported to quantify model fit.

## Results

### Study sample characteristics

Mothers were the primary caregivers for 98·0 % of children in the study. The mean age of caregivers was 27·8 (±5·48) years (Table 1). Of the 453 infants and young children, about half (48·3 %) were between the ages of 12–17·9 months, and 54·5 % were girls (Table 2).

**Table 1:** Descriptive statistics of socio-demographic and economic resource and bivariate relationship with egg consumption (*n* 453)

Variable	*n*	%	Bivariate association with egg consumption*		
			OR	95 % CI	*P*
Caregiver’s age (years)					
Mean	27·79		0·992	0·961, 1·02	0·610
sd	5·48				
Caregiver’s marital status (married)			1·11	0·491, 2·52	0·791
Caregiver’s religion					
Orthodox Christian	231	51·0	1		
Muslim	93	20·5	0·814	0·460, 1·44	0·621
Protestant/Catholic/others	129	28·5	0·796	0·453, 1·40	0·722
Caregiver’s education					
No education	106	23·4	1		
Some primary	179	39·5	2·20	1·36, 3·56	0·011
Some high school	123	27·2	1·59	0·940, 2·68	0·100
Some college or above	45	9·9	5·61	2·89, 10·9	<0·001
Caregiver’s occupation					
Unemployed	349	77·0	1		
Private sector	14	3·1	0·924	0·343, 2·49	0·868
Government employee	42	9·3	2·42	1·46, 4·03	0·001
Petty trade	31	6·8	1·87	0·975, 3·59	0·047
Self-employed	17	3·8	0·602	1·17, 2·16	0·458
Head of household education					
No education	47	10·4	1		
Some primary	158	34·9	2·81	1·32, 5·98	0·010
Some high school	152	33·5	3·99	1·87, 8·49	0·001
Some college or above	96	21·2	5·07	2·31, 11·1	<0·001
Wealth index					
Mean	0·00		1·18	1·07, 1·31	0·001
sd	2·41				
Household food insecurity access score (0 to 27)					
Mean	3·05		0·919	0·882, 0·958	<0·001
sd	5·42				
Distance to markets (min)					
Mean	18·51		1·01	0·996, 1·03	0·115
sd	13·43				
Eggs available in markets (yes)	381	84·1	2·66	1·54, 4·59	0·003
Eggs produced in the household (yes)	16	3·5	2·51	0·964, 6·52	0·118
Own chickens in the households (yes)	22	4·9	2·11	0·912, 4·89	0·096

* Egg consumption is an ordinal variable and ranges 0–5.

**Table 2:** Descriptive statistics of caregiver behaviour and child health and bivariate relationships with egg consumption (*n* 453)

Variable	Mean	sd	*n*	*%*	Bivariate association with egg consumption*		
					OR	95 % CI	*P*
Eggs purchased in the last month (yes)			294	64·9	12·7	7·38, 21·9	<0·001
Negative attitudes towards eggs (mean, 1–5)	2·78	0·79			0·627	0·427, 0·922	0·014
Positive attitude towards eggs (mean, 1–5)	3·79	0·56			1·40	0·899, 2·18	0·126
Caregiver’s self-efficacy (mean, 1–5)	3·67	0·97			1·22	0·968, 1·55	0·071
Social norm (mean, 1–5)	3·67	0·71			1·05	0·791, 1·41	0·713
Caregiver’s intent to feed eggs (1–5)	3·80	1·03			1·59	1·25, 2·02	<0·001
Positive behavioural determinants (factor score)†	0·00	1·00			1·36	1·03, 1·80	0·031
Mother’s decision making on food purchases (yes)			346	76·4	0·647	0·382, 1·10	0·101
Caregiver received information that eggs make children strong							
Never heard			251	55·4	1		
Heard from health worker			181	39·9	0·658	0·387, 1·12	0·113
Heard from radio or television			17	3·75	1·40	0·478, 4·09	0·515
Heard from someone else in the community			4	0·9	1·61	0·209, 12·4	0·702
Caregiver received information that eggs make children bright							
Never heard			275	60·7	1		
Heard from health worker			155	34·2	0·605	0·378, 0·967	0·029
Heard from radio or television			18	3·9	0·930	0·341, 2·54	0·878
Heard from someone else in the community			5	1·1	0·261	0·026, 2·68	0·274
Child sex (female)			247	54·53	1·25	0·891, 1·75	0·184
Child age (months)							
6–11·9			144	31·8	1		
12–17·9			219	48·3	2·86	1·89, 4·33	<0·001
18–23·9			90	19·9	2·55	1·46, 4·45	0·001
Food diversity (0–6, not including eggs)	2·67	1·52			1·39	1·23, 1·59	<0·001
Child’s age at egg introduction (months)	6·19	3·84			1·24	1·18, 1·31	<0·001
Child has separate food preparation (Yes)			136	30·0	0·561	0·382, 0·823	0·002
Child has own plate (yes)			392	86·5	2·04	1·13, 3·67	0·012
Child health (0–10)	6·76	1·87			1·13	1·02, 1·25	0·016
Child appetite (0–10)	6·43	1·87			1·03	0·920, 1·16	0·586
Child health and appetite (sum, 0–20)	13·19	3·23			1·05	0·986, 1·13	0·120
Fever in the last 7 d (yes)			137	30·2	0·546	0·339, 0·879	0·009
Diarrhoea in the last 7 d (yes)			55	12·1	0·859	0·534, 1·38	0·490
Cough or cold in the last 7 d (yes)			174	38·4	0·774	0·476, 1·26	0·285
Shortness of breath in the last 7 d (yes)			20	4·4	0·352	0·143, 0·864	0·015

* Egg consumption is an ordinal variable and ranges 0–5.

† Positive behavioural determinants were constructed from caregiver’s positive attitudes towards feeding eggs to children, self-efficacy, social norms and intention to feeds eggs using factor analysis assuming one factor to obtain a factor score.

### Egg consumption in infants and young children

The number of eggs consumed by children in the last 7 days ranged from 0 to 5 eggs. About half of children (53·4%) did not consume eggs, 7·1% consumed one egg, 14·8% consumed two, 11·0 % consumed three, 4·2% consumed four and 9·5% consumed five eggs, in the last 7 d. The mean number of eggs consumed in the last 7 d was higher in children 15–23·9 months of age (1·76) than children 6–14·9 months (1·03). In the last 24 h, 34·4% of children consumed eggs. The mean age of children at first introduction of eggs was 6·2 (±3·84) months (Table 2). Caregiver’s reasons for not feeding eggs to their children were not having money (31·8%), child does not like eggs (28·9%), eggs are too expensive (24·8%), child is too young (20·7%), did not think they should (12·4%), do not consume eggs in the household (9·1%) and eggs are not available in markets (3·3%).

### Socio-demographic characteristics and economic resources

Some primary education was attained by 39·5 % of caregivers and 34·9 % of the head of households. Over three-fourths of caregivers were unemployed (77·0 %) and 51·0 % were Orthodox Christians. In the bivariate models, both the caregiver’s and head of household’s education, wealth index and household food insecurity were associated with egg consumption in children. Caregivers’ report of availability of eggs in markets was positively associated with egg consumption. Neither owning chickens nor producing eggs in the household was associated with egg consumption (Table 1).

### Caregiver behaviour and child health

Around two-thirds of caregivers reported eggs were purchased in the household in the last month (64·9 %), and mothers were decision makers in food purchases among 76·4 % of households. Among caregivers who purchased eggs in the last month, caregivers were willing to spend 6·87 ETB on average (±2·25, ranges from 2 to 12) per egg, equivalent to 0·15 USD. If the price of eggs exceeds what they are willing to spend, caregivers indicated that they look for cheaper eggs (53·1 %), stop using eggs (31·6 %), replace eggs with other type of food (9·2 %) or find other solutions (6·1 %). Caregivers reported that the money paid for an egg ranged from 4·5 to 6·3 Ethiopian birr. More than half of caregivers reported they did not recognise receiving messages with information stating eggs make children strong and active (55·4 %) or eggs make children bright and sharp (60·7 %) (Table 1).

In the bivariate models, purchasing eggs and caregiver’s intent to feed eggs were positively associated, whereas caregiver’s negative attitude towards feeding eggs to children was negatively associated with egg consumption in children. Child’s age, consuming a diverse set of foods, a child’s age at the introduction to eggs and a child having their own plate were each positively associated with egg consumption (Table 2).

### Multivariable associations

In multivariable model A (Table 3), caregiver’s education, wealth index and availability of eggs in the markets were associated with egg consumption. The odds of children consuming eggs were 4·32 (*P* < 0·002) times higher when their caregivers had some college education compared with caregivers who had no education. A one-unit higher wealth index was associated with 1·13 higher odds of consuming more eggs (*P* = 0·029). A one-unit higher household food insecurity score was associated with 0·96 lower odds of consuming more eggs (*P* = 0·117). Head of household with some primary (OR, 2·09, *P* = 0·085), some high school (OR, 2·32, *P* = 0·065) and some college or higher education (OR, 2·05, *P* = 0·201) compared with no education was more likely to have children consume more eggs.

**Table 3:** Multivariable ordinal logistic regression of egg consumption on social, economic and behavioural determinants

	Model A			Model B			Model C		
	OR	95 % CI	*P*	OR	95 % CI	*P*	OR	95 % CI	*P*
Child age in months (ref: 6·0–11·9)									
12·0–17·9	2·96	1·90, 4·62	<0·001	1·95	1·23, 3·08	0·002	1·78	1·10, 2·89	0·013
18·0–23·9	3·06	1·71, 5·47	<0·001	1·62	0·774, 3·38	0·174	1·61	0·730, 3·54	0·219
Caregiver’s education (ref: not educated)									
Some primary	1·61	0·867, 3·01	0·101				1·09	0·631, 1·89	0·725
Some high school	1·22	0·630, 2·36	0·536				0·744	0·351, 1·58	0·439
Some college or higher	4·32	1·72, 10·9	0·002				2·52	1·01, 6·31	0·055
Head of household education (ref: none)									
Some primary	2·09	0·835, 5·26	0·085				2·06	0·818, 5·18	0·093
Some high school	2·32	0·881, 6·11	0·065				2·30	0·863, 6·12	0·074
Some college or higher	2·05	0·638, 6·57	0·201				1·73	0·519, 5·77	0·353
Wealth index	1·13	1·01, 1·26	0·029				1·01	0·900, 1·14	0·848
Household food insecurity (0–27)	0·961	0·916, 1·01	0·117				1·00	0·940, 1·07	0·983
Egg availability in markets (ref: no)	3·25	1·65, 6·39	0·001				0·968	0·492, 1·91	0·930
Eggs purchased in the last month (ref: no)				9·73	5·63, 16·8	<0·001	8·94	5·00, 16·0	<0·001
Negative attitude towards eggs (0–5)				0·801	0·571, 1·12	0·197	0·809	0·564, 1·16	0·244
Positive behavioural determinants (factor score)*				1·37	1·09, 1·72	0·005	1·37	1·08, 1·72	0·006
Food diversity (0–6, not including eggs)				1·17	1·03, 1·33	0·010	1·15	1·00, 1·33	0·034
Child’s age at egg introduction (months)				1·22	1·14, 1·30	<0·001	1·22	1·15, 1·30	<0·001
Child’s has own plate (ref: no)				2·79	1·41, 5·53	0·001	2·78	1·36, 5·71	0·002
Child health and appetite (0–20)				1·06	1·01, 1·13	0·030	1·06	1·00, 1·12	0·039
Fever (ref: no)				0·672	0·399, 1·31	0·118	0·678	0·398, 1·16	0·126
Model fit (C-statistic)	0·680			0·797†			0·799†		

Model A included only the socio-demographic characteristics and economic resources. Model B included only caregiver behaviour and child health. Model C included both sets of variables.

* Positive behavioural determinants were constructed from caregiver’s positive attitudes towards feeding eggs to children, self-efficacy, social norms and intention to feeds eggs using factor analysis assuming one factor to obtain a factor score.

† Model explained about three-fifths of the variability that could have been explained.

In multivariable model B (Table 3), purchasing eggs was associated with children’s consumption of eggs (OR, 9·73, *P* < 0·001), as was positive behavioural determinants (OR, 1·37, *P* = 0·005). Consuming more diverse foods (OR, 1·17, *P* < 0·010), being older in age at introduction to eggs (OR, 1·22, *P* < 0·001), having their own plate (OR, 2·79, *P* = 0·001) and ranking higher in health and appetite (OR, 1·06, *P* < 0·030) were each positively associated with consuming more eggs. History of having a fever in the last 7 d was associated with consuming fewer eggs (OR, 0·67, *P* = 0·118).

In model C (Table 3), the OR of caregiver education, wealth index, household food insecurity and egg availability at markets were highly attenuated compared with model A. Associations of caregiver behaviours and child health with egg consumption in model C were like those in model B.

We found no statistical interactions of socio-demographic characteristics and economic resources with caregiver behaviours and child health (results not shown). These results mean that caregiver behaviour and child health in this sample were associated with egg consumption at all levels of socio-demographic characteristics and economic resources. The sensitivity analysis using egg consumption in the past 24 h as an outcome produced similar findings as shown for the ordinal egg consumption.

## Discussion

The study aimed to identify determinants of egg consumption in infants and young children aged 6–23·9 months in Ethiopia. Wealth, household food insecurity, education of caregiver and household head, and availability of eggs in markets were the socio-demographic and economic determinants associated with egg consumption. Caregiver’s purchasing of eggs, positive behavioural determinants, child food diversity, child having own plate, the child’s age at introduction to eggs, child history of fever in the last 7 d, and child health and appetite were associated with egg consumption. The associations of socio-demographic characteristics and economic resources with egg consumption were mainly mediated through caregiver behaviour and child health (i.e. caregiver’s rating of overall child health). Although the findings need to be further corroborated by longitudinal observational studies and intervention trials, the findings highlight those mitigating economic constraints, increasing availability of eggs in households and modifying caregivers’ behaviour can be used by interventions for which the aim is to improve egg consumption among infants and young children.

The prevalence of egg consumption reported in this study (34·4 %) is higher than previously reported in Ethiopia DHS 2016 (prevalence ranges 12·5–16·1 %) for the same age group and in Asian countries (28·9 %) but is lower than the prevalence of egg consumption seen in Latin American (37·2 %)^(4,24)^. Increasing egg consumption has the potential to meet nutritional needs, promotes growth and improves cognitive function, physical activity and general health of infants and young children^(3,8)^, thus increasing consumption of eggs is needed. In addition to economic constraints, caregivers reported that their main reasons for not feeding eggs were not knowing they need to do so and believing the child is too young for eating eggs^(8,12)^. Caregivers concern to introduce eggs to younger children aged 6–12 months included fear food allergies and difficulty in digestion^(12)^. These views of caregivers suggest gaps in caregivers’ knowledge about appropriate age to initiate feeding eggs in infant and young children. Diffusion of nutrition information has been previously shown to improve infant and young child feeding practices in Bangladesh^(25)^. Thus, promoting eggs among caregivers may potentially improve feeding and consumption of eggs. Information from a campaign promoting eggs would be expected to enter caregivers’ social networks and result in behaviour change^(25)^.

Caregivers’ positive behaviour to feed eggs in this study encompassed caregiver’s positive attitudes towards feeding eggs to children, self-efficacy to feed eggs, social norms and intention to feed eggs. Using a single variable allowed efficient examination of the association between multiple aspects of caregivers’ behaviour towards eggs and egg consumption. The finding that caregivers’ positive behaviour is related to egg consumption indicates that future intervention studies should target caregivers’ attitudes towards feeding eggs to children, self-efficacy to feed eggs, social norms and intention to feed eggs.

Caregivers’ positive behaviour to feed eggs to children can only improve egg consumption when eggs are available in the household. Only 3·5 % of household reported egg production and about two-thirds reported purchasing eggs in the last month. Homestead egg production has potential to address undernutrition through incorporating eggs in child diets^(26)^. Development of feasible and sustainable programmes to improve the availability of eggs in households is needed.

Caregiver’s literacy has been previously shown to influence child nutrition and child development^(27,28)^. This study has further shown that caregiver’s education (and that of the household head) is associated with egg consumption in infants and young children. Household wealth and household food insecurity were both associated with egg consumption in the expected direction. In addition, caregivers explained that financial concerns and cost of eggs were the main reasons for not feeding eggs. In line with these findings, a previous study on egg consumption in women and children showed that egg consumption is strongly related to socio-economic status in a dose–response fashion^(7)^. A review of qualitative studies that examined barriers of adequate infant and young child feeding in lower income countries has shown that caregivers reported replacement of higher quality foods with lower quality foods due to financial reasons or drought, indicating household food insecurity^(26)^. In the current study, most caregivers (53·1 %) reported they will look for cheaper eggs and a third of the caregivers reported they will stop using eggs, if the price of eggs exceeds what they are willing to spend. In addition, implementing nutritional programmes that provide food, free of cost in conjunction with behavioural change communication, has been shown to improve household food choices^(29)^. From a randomised controlled trial in Burkina Faso, a culturally tailored behavioural change intervention that provided messages to women and community leaders and provision of chickens increased egg consumption in children^(15)^. Thus, improving household economic resources may be important to enhance egg consumption in infants and young children.

Caregiver’s negative and positive attitudes towards feeding eggs, self-efficacy, intent to feed eggs and social norms were related to egg consumption in infants and young children. Attitudes, norms and self-efficacy are among the prominent constructs theorised to be important determinants of behaviour change^(18)^. Previous qualitative studies have shown that caregiver’s perception, financial decision making and social factors can either inhibit or enhance meeting recommended child feeding practices^(26,30)^. A qualitative study in Ecuador found that caregivers considered eggs to be healthy and nutritious but heavy for infant stomachs^(12)^. Thus, nutritional interventions that aim to improve egg consumption need to address caregiver’s attitudes, and culturally informed practices that inform decision making.

Infants and young children who consumed more diverse foods were likely to have higher egg consumption. Controlling for food diversity (with exclusion of eggs) in the multivariable models means that the other determinants of egg consumption were important beyond their influence on child food diversity.

The association of socio-demographic characteristics and economic resources with egg consumption may be mediated through caregiver behaviour and health variables. Households with greater wealth may be more likely to buy eggs, to have children fed from their own plate and have more positive attitudes towards feeding eggs, which in turn may result in higher egg consumption. Similarly, a caregiver’s education may influence egg purchases, negative attitudes towards eggs and food diversity that in turn determines egg consumption. A better understanding of these mechanisms is warranted using longitudinal studies.

Conclusion on causes of egg consumption cannot be reached given the cross-sectional design of the study. Nevertheless, the findings contribute to providing groundwork for future intervention studies. Information provided by caregivers through recall can result in misclassification on the number of eggs a child was fed. In the sensitivity analysis, we examined egg consumption in the past 24 h in addition to egg consumption in the last 7 d and obtained similar results. The current study may be limited in capturing seasonal variation in availability of eggs in markets and consequently egg consumption.

In conclusion, slightly more than half of infants and young children aged 6–23·9 months did not consume eggs. Egg consumption was higher when caregivers and household heads had higher education, economic resources were better and eggs were available in markets and purchased. The influence of these determinants on egg consumption may operate through caregiver behaviour and child health. Interventions are needed to overcome constraints related to education, economic resources, availability of eggs in households and caregiver’s behaviour to promote egg consumption for infants and young children.

## Supplementary material

For supplementary material accompanying this paper visit https://doi.org/10.1017/S1368980022001112. click here to view supplementary material
