# Supplementary material for: Determinants of egg consumption by infants and young children in Ethiopia
Source: Public Health Nutr. 2022 May 16;25(11):3121–30. doi: 10.1017/S1368980022001112 (PMC9991633; doi:10.1017/S1368980022001112)
Supplement: Supplementary file 1 [file S1368980022001112sup.zip › S1368980022001112sup001.pdf]

Supplementary material

ETHIOPIA BASELINE QUESTIONNAIRE – 2019				
Teamid	Team identifier			
Intid	Interviewer identifier			
Rgn	Region		Oromia ...1<br>Snnp ... 2	
Twn	Town	**oromia:**<br>Shashemene .01<br>Arsi negele .02<br>Jimma.03<br>Haromya.04	**Snnp**<br>Aleta wendo 05<br>Dila .06<br>Tepi.07<br>Arbaminche.08	
Kebname	Kebele name			
Kebid	Kebele identifier			
Eaid	Ea identifier			
Hhid	Household identifier (from cluster control form)			
Hhlat	Household latitude (gps)<br>Must take the gps at hh		N __ __ . __ __ . __ __ __	
Hhlon	Household longitude (gps)<br><br>Must take the gps at hh		E __ __ __ ° __ __ __ __ __	
Date	Visit 1<br><br>__ __ / __ __/2019 ec	Visit 2<br><br>__ __ / __ __/2019 ec	Final visit: __ __ / __ __ / 1 9<br>*dd* *mm* *yy*	
Result	☐	☐	Final result: ☐	
Start	Start time of interview		Hh:mm __ __ : __ __	
End	End time of interview		Hh:mm __ __ : __ __	
Final result codes:			Refused 4	

Completed ..1<br>No household member or no competent/right respondent<br>At home at time of visit .... 2<br>Entire household absent for long period or moved away 3	Dwelling vacant / address not a dwelling .... 5<br>Dwelling destroyed .... 6<br>Dwelling not found .... 7<br>Other (specify).. 8
Enumerator's	Name
	Signature
Supervisor: check through questionnaire and have any corrections made, then complete and sign below	
Supervisor's	Name
	Signature

LANGUAGE OF QUESTIONNAIRE**	0 1	LANGUAGE OF INTERVIEW**		NATIVE LANGUAGE OF RESPONDENT**		TRANSLATOR USED (YES = 1, NO = 2)	
LANGUAGE OF QUESTIONNAIRE**	ENGLISH	**LANGUAGE CODES:	01 ENGLISH<br>02 AMHARIC	03 TIGRIGNA<br>04 OROMIFFA	05 LANGUAGE 5<br>06 LANGUAGE 6		

Module 1: HOUSEHOLD COMPOSITION

We would like some information about the people who usually live in your household. Please include all family and non-family members (such as domestic servants, lodgers, or friends) who usually live together in the same dwelling and eat from the same pot of food. A member of the household must have lived in the household for at least 6 of the past 12 months. Start with **the head of the household.** ***Start by listing the head of the household, the respondent, the spouse to the head of the household (if applicable), any other adult, and finally, children.***

					ONLY for persons aged < 5 years					***Only for head of household***		
Line no. (*hh_ pid*)	Name of household member (*hh_a*)	What is [name]'s relationship to the head? (*hh_rel*)	What is [name]'s gender? (*hh_b*)	How old is [name] in completed years? *Please indicate age in years* (*hh_ca*)	How old is [name] in completed months? *Please indicate age in months (hh_cb)*	On what day, month and year was ___ born?	Who is [name]'s main caregiver? *Record line number of caregiver* (*hh_carg*)	Who is [name]'s mother? Dead…21 Living elsewhere …22	Who is [name]'s father? Dead…21 Living elsewhere …22	Has **[name]** ever attended school?	What is the highest level of school **[NAME]** has attended?	What is the highest class/year **[NAME]** completed at that level?
		Head……..…1<br>Spouse….….2<br>Son/daughter.3<br>Son/daughter in-la………...4<br>Grandchild....5<br>Parent………6<br>Parent in-law…………7<br>Brother/sister …………….8<br>Auntie/uncle… …………….9<br>Nephew/ niece………10<br>Grandparent..... ....11<br>Brother/sister in-law…….12<br><br>Other relative of HH head or	Male… ………1<br><br>Female.. 2	----- Years	----Months	Day _ _<br><br>Month _<br><br>Year _ _				Yes…1<br>No…2	Completed first year…1<br>Put number of highest completed class. For example, if left before completing third grade, put 2 as second grade is last completed. completed<br><br>High School.12<br><br>Technical or Vocational Training…….13<br><br>University/ College diploma 14	

		spouse of head............13<br>Domestic help or related to domestic help………14<br><br>Not related to HH head or spouse of the head…….15<br><br>Other…...16<br>Specify									University/ College degree or higher .... 15<br>**N_A……9_8**<br>**D_K_….8_8**	
01	Head of household	[see above options]	M….1<br>F….2			DD/MM /YY ……/… …/…...	☐ ☐					
02	Caregiver	[see above options]	M….1<br>F….2			DD/MM /YY ……/… …/…...	☐ ☐					
03	Child 1 (6-59 month)	[see above options]	M….1<br>F….2			DD/MM /YY ……/… …/…...	☐ ☐					
04	Child 2(6-59 month)	[see above options]	M….1<br>F….2			DD/MM /YY ……/… …/…...	☐ ☐					

	**MODULE 2: CAREGIVER CHARACTERISTICS**			
N°	QUESTIONS	answers	CODES	SKIPS
2.1	Identification of respondent from household roster	identification from hh roster	☐☐	
2.2	What is your relationship with [CHILD NAME]?	mother	1	
		grandmother/father	2	
		father	3	
	CIRCLE ONLY ONE CHOICE.	other: ______________________	8	
2.3	What is your level of education? *CIRCLE ONLY **ONE** CHOICE.*	none/illiterate	1	→ 2.5
		Informal education (Read and write)	2	→ 2.5
		Formal Education	3	
2.4	What is the highest level of school **[NAME]** has attended?			
2.5	What is your main occupation?	attends to home chores	01	
		agriculture – own farm	02	
		agriculture – works on someone else's farm	03	
	*CIRCLE ONLY **ONE** CHOICE*	employed in private sector	04	
		government employee/ civil service	05	
		petty trade/	06	
		selfemployeed	07	
		student	10	
		unemployed	11	
		other: ______________________	88	
2.6	What is your current marital status?	married/ living together	1	
		single	2	
	*CIRCLE ONLY **ONE** CHOICE.*	divorced/ separated	3	
		widowed	4	
2.7	What is your religion?	Orthodox……………………….1<br>Protestant……………………….2<br>Catholic…………………………3<br>Muslim………………………….4<br>Other……………………………5		

HOUSEHOLD CHARACTERISTICS AND ASSETS		
2.8	Does your household have electricity?	Yes 1 No 0
2.9	What fuel does your household mainly use for cooking?	1 Electricity<br>2 LPG/cylinder<br>3 Natural Gas<br>4 Biogas<br>5 Kerosene stove<br>6 Coal / Lignite<br>7 Charcoal<br>8 Firewood<br>9 Straw / Shrubs / Grass/ Sawdust<br>10 Agricultural crops<br>11 Animal dung<br>12 No food cooked in household<br>13 Don’t know<br>96 Other (specify)
2.10	What is the main material of the floor of the dwelling?	1 Earth / sand<br>2 Dung<br>3 Wood planks<br>4 Palm / bamboo<br>5 Parquet / polished wood<br>6 Vinyl / asphalt strips<br>7 Ceramic tiles<br>8 Cement<br>9 Carpet<br>96 Other (specify)
2.11	What is the main material of the roof of the dwelling?	1 No roofing

			2 Thatch / palm leaves<br>3 Sod<br>4 Rustic mat<br>5 Palm / bamboo<br>6 Wood planks<br>7 Cardboard<br>8 Metal/zinc<br>9 Wood<br>10 Calamine / cement fiber<br>11 Ceramic tiles<br>12 Cement<br>13 Roofing shingles<br>96 Other (specify)
2.12	What is the main material of the exterior walls of the dwelling?		1 No walls<br>2 Cane/palm/trunks<br>3 Dirt/Mud<br>4 Bamboo and mud<br>5 Stone and mud<br>6 Uncovered adobe<br>7 Plywood<br>8 Cardboard<br>9 Reused wood<br>10 Cement<br>11 Stone with lime/cement<br>12 Bricks<br>13 Cement blocks<br>14 Wood planks/shingles<br>15 Covered adobe<br>96 Other (specify)

2.13	Now I'm going to ask if you or your household owns any of the following items.	

Do you or anyone in your household own a …?	Yes	No
Radio	1	0
Television	1	0
Mobile Telephone	1	0
Non-mobile telephone	1	0
Wristwatch	1	0
Bicycle	1	0
Motorcycle, scooter, auto rickshaw	1	0
Car or truck	1	0
Computer	1	0
Animal-drawn cart	1	0
Boat with a motor	1	0
Fan	1	0
Electric iron	1	0
Refrigerator	1	0
Dish washer/washing machine	1	0
Air conditioner	1	0
Generating set	1	0
Cable TV	1	0

2.14	Does any member of your household own any agricultural land?	Yes…. 1<br>No…. 0 ……………**skip to Q.2.16**
2.15	If so, how many acres?	………..
2.16	Does this household own any livestock, herds, other farm animals, or poultry?	Yes…. 1<br>No…. 0 ……………. **skip to Module 3**
2.17	How many [animal] does the household own?	
	2.10a. Cows/Bulls	_______
	2.10b. Other cattle	_______
	2.10c. Horses/Donkeys/Mules	_______
	2.10d. Goats	_______
	2.10e. Sheep	_______
	2.10f. chicken - cock/broiler	_______
	2.10g. chicken - hens/layers	_______

	2.10h. pullets and day-old-chicks	________
	2.10j. Other specify	________

Module 3: HEN Assets		
3.1	Do you have chicken - hen/layers?	1 Yes<br>0 No………. **Skip to Q.3.9**
3.2	How many chicken - hen/layers do you have?	1 1 to 9<br>2 10 to 29<br>3 30 or more
3.3	Has any hen/layers in household produced any eggs in the past ONE month?	
3.4	How many clutching periods did hen/layers have on average in the last SIX months?	
3.5	How many eggs per clutching did hen/layers lay on average before the last clutching period?	
3.6	How many hen/layers had their clutching period in the past 3 months?	
3.7	Did this household sell the eggs in the last 3 months?	1 Yes<br>0 No **Skip to** Q.3.9
3.8	How many of the eggs produced did you sell in the last 3 months?	
**Bank account and number of Rooms**		
3.9	Does any member of this household have a bank account?	1 Yes<br>0 No<br>98 Don’t know
3.10	How many rooms are there in total in your household?	
3.11	How many rooms are used for sleeping in your household?	

MODULE 4: WASH, SANITATION, AND HYGIENE (WASH)			
N°	QUESTIONS	ANSWERS	CODES
4.1	What is the **main** water source used by the members of this house, **drinking water**?<br><br>*CIRCLE ONLY **ONE** CHOICE.*	Piped water inside the house	01
		Piped water in the compound	02
		Public tap/standpipe	03
		Protected dug well	04
		Protected spring	05
		Rainwater collection	06
		Tanker	07
		Unprotected spring	08
		Unprotected dug well	09
		Small water vendor	10
		Tanker truck	11
		Bottled water	12
		River / stream/lake/lagoon water	13
		Other: _______________ water	88
		Don't know	9
4.2	Where is that water source located?	In own dwelling	1
		In own yard/plot	2
		Outside the yard, on another place	3
		Other: ____________________________	8
4.3	How long it takes to get to the source, take water and return home?	Minutes	☐☐☐
		Don't know (999)	
4.4	What type of toilet facility is usually **used** here at home?<br><br>*The question is about the bathroom they **use,** not the type they **have.***<br><br>*CIRCLE ONLY **ONE** CHOICE.*	Flush to piped sewer system	01
		Flush to septic system	02
		Pour-flush to pit	03
		Vip/simple pit latrine with floor/slab	04
		Composting/dry latrine	05
		Flush or pour-flush elsewhere	06
		Pit latrine without floor/slab	07

		Service or bucket latrine	08
		Hanging toilet/latrine	09
		No facility, field, bush, plastic bag	10
		Other: ______________________________	88
4.5	Is the toilet facility shared with other households?	BATHROOM SHARED	YES 1<br>NO 0

MODULE 6: CHILD HEALTH AND APPETITE

Now I would like to ask you questions about **([child name 1 &/or 2])** health and appetite.

6.1. Has **([child's name 1 &/or child 2])** had [SYMPTOM] in the past 7 days?

	Child name 1			Child name 2		
	Yes	No	IDK	Yes	No	IDK
Fever	1	0→**6.3**	98→**6.3**	1	0→**6.3**	98→**6.3**
Cough/Cold	1	0→**6.3**	98→**6.3**	1	0→**6.3**	98→**6.3**
Fast breathing or shortness of breath?	1	0→**6.3**	98→**6.3**	1	0→**6.3**	98→**6.3**
Diarrhea	1	0→**6.3**	98→**6.3**	1	0→**6.3**	98→**6.3**

6.2 When **child's name 1 &/or child 2])** had [SYMPTOM] Did you give less to eat than normal, just about the same amount, more than usual or nothing to eat at all?

	**Child name**<br>Yes=1 No=0	**Child name 2**<br>Yes=1 No=0
Much less		
A little bit less		
About the same amount		
More		
Stopped giving food		
Do not know		

6.3 When you compare (([child's name 1 &/or child 2] with the children of the area of his/her age how would you rank the (([child's name 1 &/or child 2] health
Ask the mother to put on the line: show card

1	2	3	4	5	6	7	8	9	10
Health is not good				Health is good			Health is very good		

6.4 On a normal day (child's name 1 &/or child 2] appetite

1	2	3	4	5	6	7	8	9	10
appetite is not good				Appetite is good			appetite is very good		

Module 7: CHILDREN FEEDING PRACTICES				
N°	QUESTIONS	ANSWERS	CODES	
7.1	Has (child name) ever been breastfed?<br><br>*INCLUDE GIVING BREAST MILK BY SPOON OR BOTTLE OR BREASTFEEDING BY OTHER WOMEN.*	Yes 1<br>No 0<br>DK 98		
7.2	When you are making (child name's) food, is it just the same as the food for the rest of the family?	Yes 1 -**SKIP TO Q.**7.4<br><br>No 0<br>DK 98		
7.3	How is the food for (child name) different?<br><br>*DO NOT READ THE ANSWERS.*<br>*CHECK ALL APPLICABLE ANSWERS.*	Make foods softer	1	
		Make foods less spicy	2	
		Add special ingredients/use special recipe	3	
		Others: ______________	4	
		Don't know	98	
7.4	Does (child name) usually eat on a plate or bowl just for him or her?	Child eats from own plate	1	
		Child eats from shared plate, but child's food is separated for him or her	2	
		Child eats from shared plate with others	3	
		Other<br>________________________	4	
7.5	Who usually feeds (child name)?	The child eats alone	1	
		Mother	2	
		Father/husband	3	
		Grandmother/father	4	
		Older brother/sister	5	
		Nanny	7	
		Other specify<br>________________________	8	

7.6	Was (child name) breastfed during the day or night yesterday?	Yes 1<br>No 0<br>DK 98		
7.7	Sometimes babies are breastfed with breast milk in different ways, for example with a spoon, cup or feeding bottle. That happens when the mother cannot stay with the baby at all times. Sometimes the babies are breastfed by another woman, or the breast milk from another woman is given to the baby through a spoon, cup or FEEDING BOTTLE, or any other way. That can happen when a mother cannot breastfeed her own child.<br><br>Did (name of the child) drink breast milk in any of these forms during the day or night yesterday?	Yes 1<br>No 0<br>DK 98		

7.8	Now I would like to ask you about liquids or foods that ([childsname]) had yesterday during the day or at night. I am interested in whether your child had the item I mention even if it was combined with other foods. Did ([childsname]) drink or eat: Did (name) have any (item from the list)?:<br><br>*Read the list of liquids starting with 'Plain Water' Ask the mother to mention and tick from the list*	Yes	No	Don't know
	**Plain water?**	A	0	98
	**Infant formula such as nan, *FRANCE LAIT, LIPTO MIL, S-26, PROMIL* in tins or sachets?**	1b	0	98
	**Cow milk (such as tinned, powdered) or fresh animal milk?**	1c	0	98
	**Juice or juice drinks?**	1d	0	98
	**Clear broth (rice water, boiled meat water)?**	1e	0	98
	**Thin porridge (atmit)?**	1f	0	98
	**Any other liquids such as tea?**	1g	0	98
	**Ors (oral rehydration solution)?**	1h	0	98

	**Yogurt (including locally made)?**	1i	0	98
	**Bread, rice, noodles, pasta(macaroni), injera, kita, nefro or other foods made from grains**	1j	0	98
	**Fortified baby food such as Cerifam, Faffa or Cerelac.**	1k	0	98
	**Pumpkin, carrots, squash, or sweet potatoes that are yellow or orange inside**	1l	0	98
	**White potatoes, white yams, enset (false banana), cassava, or any other foods made from roots**	1m	0	98
	**Any dark green leafy vegetables like gomen, spinach, swiss chard, kale or amaranth leaves**	1n	0	98
	**Ripe mangoes, ripe papayas**	1o	0	98
	**Any other fruits or vegetables (cactus fruit, banana, orange, watermelon, apple, tomato)**	1p	0	98
	**Liver, kidney, heart, or other organ meats?** *If "Yes", confirm with respondent that child actually consumed meats and not just sauce cooked with meat. If only sauce consumed, mark "No".*	1q	0	98
	**Any meat, such as beef, pork, lamb, goat, chicken, or duck?** *If "Yes", confirm with respondent that child actually consumed meats and not just sauce cooked with meat. If only sauce consumed, mark "No".*	1r	0	98
	**Chicken Eggs?**	1s	0	98
	**Other types of eggs?**	1t	0	98
	**Fresh or dried fish?** *If "Yes", confirm with respondent that child actually consumed fish etc, and not just sauce cooked with fish etc. If only sauce consumed, mark "No".*	1u	0	98
	**Any foods made from beans, peas, chickpea, lentils, or pulses?**	1v	0	98
	**Any nuts or seeds such as peanuts, sesame, sunflower seeds?**	1w	0	98
	**Cheese, yogurt, or other milk products?**	1x	0	98
	**Any oil, fats, or butter, or foods made with any of these?**	1y	0	98
	**Any sugary foods such as chocolates, sweets, candies, pastries, cakes, or biscuits?**	1z	0	98
	**Any savoury foods such as crisps, chips, salted biscuits or instant noodles?**	1aa	0	98

	**Condiments for flavour, such as pepper, hot pepper, onions, spices, herbs, or fish powder?**	1bb	0	98
	**Any sweetened drink such as soft drinks, fizzy drinks, soda or chocolate drinks?**	1cc	0	98
	**Any purchased food**<br>**Check the ingredient and code the right section above and write the name of the food here** ____	1dd	0	98
**7.9**	Did (*NAME*) eat solid or semi-solid (soft, mushy) food yesterday (e.g. soft cooked rice, cooked potatoes), during the day or night?	Yes 1<br>No 0 → **SKIP TO Q**.7.12<br>DK 98		
**7.10**	How many times did (*NAME*) eat solid or semi-solid (soft, mushy) food yesterday (e.g.**injera, kita, nefro, porridge...,**), during the day or night?	______times		
**7.11**	Yesterday, during the day or night, did *(name)* consume any lipid based nutrient supplement like plumpy nut?	Yes 1<br>No 0<br>DK 98		

MODULE 8: DECISIONS ABOUT FOOD AND DECISIONS ON PURCHASE		
8.1	Please indicate who mainly makes the decisions about purchasing of [Fruits and vegetables]	1 Head of Household<br>2 Self<br>3 Both head of household and respondent<br>4 Someone else<br>5 Decision made by 2 or more members of the household, excluding respondent<br>6 Decision made by 2 or more members of the household, including respondent<br>7 Item is not purchased in the household<br>98 DK
8.2	Please indicate who mainly makes the decisions about purchasing of [Rice, beans, lentils and other legumes, etc.]	1 Head of Household<br>2 Self<br>3 Both head of household and respondent<br>4 Someone else<br>5 Decision made by 2 or more members of the household, excluding respondent<br>6 Decision made by 2 or more members of the household, including respondent<br>7 Item is not purchased in the household<br>98 DK
8.3	Please indicate who mainly makes the decisions about purchasing of [Meat, fish, poultry, etc.]	1 Head of Household<br>2 Self<br>3 Both head of household and respondent<br>4 Someone else<br>5 Decision made by 2 or more members of the household, excluding respondent

		6	Decision made by 2 or more members of the household, including respondent
		7	Item is not purchased in the household
		98	DK
8.4	Please indicate who mainly makes the decisions about purchasing of [Eggs]	1	Head of Household
		2	Self
		3	Both head of household and respondent
		4	Someone else
		5	Decision made by 2 or more members of the household, excluding respondent
		6	Decision made by 2 or more members of the household, including respondent
		7	Item is not purchased in the household
		98	DK
8.5	Please indicate who mainly makes the decisions about purchasing of [Packaged food products (spaghetti, Macaroni, snacks, etc.)]	1	Head of Household
		2	Self
		3	Both head of household and respondent
		4	Someone else
		5	Decision made by 2 or more members of the household, excluding respondent
		6	Decision made by 2 or more members of the household, including respondent
		7	Item is not purchased in the household
		98	DK
8.6	Please indicate who mainly makes the decisions about purchasing of [Special foods for children]<br><br>Special foods for children are those foods that are	1	Head of Household
		2	Self
		3	Both head of household and respondent

	bought only for children under the age of 5, such as cerifam, Faffa etc.<br>No other members of the household consume that food.	4 Someone else<br>5 Decision made by 2 or more members of the household, excluding respondent<br>6 Decision made by 2 or more members of the household, including respondent<br>7 Item is not purchased in the household<br>98 DK
8.7	Where foods for the household are mostly purchased (type of outlets)?	1 Neighbor shop<br>2 Retail market<br>3 Wholesale market<br>4 Minimarket/ Supermarkets<br>5 Street vendors<br>6 Indoor trader (door to door)<br>96 Other (specify)
8.8	How long does it take to get there by most common means of transportation? (in minutes)	____ minutes
8.9	How frequently is [int_out] visited?	1 Daily<br>2 Two to six times/week<br>3 Once/week<br>4 Once/two weeks<br>5 Once/month<br>6 Less than once/month
8.10	Does [int_out] have egg available for purchase?	1 Yes<br>0 No<br>98 Don't know
WILLINGNESS TO PAY FOR EGGS		
8.11	Have you purchased chicken eggs during the last 30 days?	1 Yes<br>0 No → **skip to Module 9**
8.12	The last time you bought eggs, where did you buy it?	1 Neighbor shop<br>2 Retail market

		3 Wholesale market<br>4 Minimarket/ Supermarkets<br>5 Street vendors<br>6 Indoor trader (door to door)<br>96 Other (specify)
8.13	The last time you bought eggs how much did you pay per egg?	_______ ETB
INTRODUCTION - I would now like to ask you some questions about potential changes to the prices of chicken eggs. In answering these questions, please keep in mind the following: a. assume that your income will stay the same even if egg prices change, b. alternatives exist for chicken eggs such as other types of eggs or foods.		
8.14	You said that you last bought an egg for [8.13]. If the price increased by **1 ETB** to [calc_8.13], would you continue to buy eggs?	1 Yes<br>0 No → **skip to Q. 8.16**
8.15	If the price increased to [calc_81.14 +1 EBT] would you continue to buy eggs?	1 Yes → **skip to Q. 8.17**<br>0 No
8.16	If the price increased to [calc_8.14 +0.5 EBT], would you continue to buy eggs?	1 Yes<br>0 No
8.17	What is the maximum price you would be willing to pay for an egg?	________ETB
8.18	If the price of eggs at the [calc_10.7] exceeds what you would be willing or able to pay, what would you do?	1 Look for cheaper eggs somewhere else<br>2 Replace chicken eggs with other type of eggs<br>3 Stop using them<br>96 Other (specify)

Module 9: Faffa consumption patterns

9.1 Has (childnames) ever consumed Faffa?

**Child name**	**Yes**	**No**
Child name 1	1	0 →**skip Q.9.6**
Child name 2	1	0 →**skip Q..9.6**

9.2 When was the last time that (childname) consumed Faffa?

	**Child name 1**		**Child name 2**	
Yesterday	1 →**skip Q.9.4**	0	1 →**skip Q.9.4**	0
During the past 7 days	1 →**skip Q.9.4**	0	1 →**skip Q.9.4**	0
Less than a month ago	1 →**skip Q.9.4**	0	1 →**skip Q.9.4**	0
More than a month ago	1	0	1	0

9.3 What are the reasons why (childname) consumed Faffa more than a month ago?

	**Child name 1** Yes=1 No=0	**Child name 2** Yes=1 No=0
Not available		
Expensive		
Not needed		
Homemade food is better		
Don't think it is good		
Not aware of the product		
Other (specify)…		

9.4 How often do you give Faffa to (childname)?

	**Child name 1** Yes=1 No=0	**Child name 2** Yes=1 No=0
Everyday		
Every other day		
2 or 3 times per week		
One time per week		
Less than one time per week		
Other(specify)		

9.5 What are the main reasons why you give Faffa to (NAME)? Mark all mentioned

	**Child name 1** Yes=1 No=0	**Child name 2** Yes=1 No=0
Affordable		
Available		
Preferred by child		
Good for child's health		

<table>
<tr><td></td><td></td><td><table>
<tr><td>Good for child’s strength</td><td></td><td></td></tr>
<tr><td>Advised by health worker</td><td></td><td></td></tr>
<tr><td>Advised by family/friends/neighbours</td><td></td><td></td></tr>
<tr><td>Easy to prepare</td><td></td><td></td></tr>
<tr><td>Has vitamins and/or minerals</td><td></td><td></td></tr>
<tr><td>No time to prepare porridge flour at home</td><td></td><td></td></tr>
<tr><td>Promotion/advertised</td><td></td><td></td></tr>
<tr><td>Other (specify)</td><td></td><td></td></tr>
</table></td></tr>
<tr><td>9.6</td><td>What are the reasons why (childname) has never consumed Faffa? Mark all mentioned</td><td><table>
<tr><td></td><td>Child name 1
Yes=1 No=0</td><td>Child name 2
Yes=1 No=0</td></tr>
<tr><td>Not available</td><td></td><td></td></tr>
<tr><td>Expensive</td><td></td><td></td></tr>
<tr><td>Not needed</td><td></td><td></td></tr>
<tr><td>Homemade food is better</td><td></td><td></td></tr>
<tr><td>Don’t think it is good</td><td></td><td></td></tr>
<tr><td>Not aware of the product</td><td></td><td></td></tr>
<tr><td>Other (specify)…</td><td></td><td></td></tr>
</table></td></tr>
<tr><td>9.7</td><td>Did you ever bought Faffa ?</td><td>Yes …1
No….0 →skip Q.9.11</td></tr>
<tr><td>9.8</td><td>The last time that you bought Faffa, where did you buy it?</td><td>Community shop/kiosks…1
Retail market…2
Wholesale market…3
Mini/Supermarkets…4
Street vendors…5
Indoor trade (door to door) …6
Open market ….7
Other (specify)…8</td></tr>
<tr><td>9.9</td><td>The last time that you bought Faffa, what type of package did you buy?</td><td>SHOW OPTIONS
200 g sachet…1
200 g box…2
300 g sachet…3
300 g box…4</td></tr>
</table>

<table>
<tr><td></td><td></td><td>500 g sachet…5
Don't know/don't remember…6</td></tr>
<tr><td>9.10</td><td>The last time that you bought Faffa, how much did you pay for it?</td><td>_____ ETB</td></tr>
<tr><td colspan="3">Cerifam consumption patterns</td></tr>
<tr><td>9.11</td><td>Has (childname) ever consumed Cerifam?</td><td><table><tr><th>Child name</th><th>Yes</th><th>N0</th></tr><tr><td>Child name 1</td><td>1</td><td>0 →skip Q.9.16</td></tr><tr><td>Child name 2</td><td>1</td><td>0 →skip Q..9.16</td></tr></table></td></tr>
<tr><td>9.12</td><td>When was the last time that (NAME) consumed Cerifam?</td><td><table><tr><th></th><th colspan="2">Child name 1</th><th colspan="2">Child name 2</th></tr><tr><td>Yesterday</td><td>1 →skip Q.9.14</td><td>0</td><td>1 →skip Q.9.14</td><td>0</td></tr><tr><td>During the past 7 days</td><td>1 →skip Q.9.14</td><td>0</td><td>1 →skip Q.9.14</td><td>0</td></tr><tr><td>Less than a month ago</td><td>1 →skip Q.9.14</td><td>0</td><td>1 →skip Q.9.14</td><td>0</td></tr><tr><td>More than a month ago</td><td>1</td><td>0</td><td>1</td><td>0</td></tr></table></td></tr>
<tr><td>9.13</td><td>What are the reasons why (childname) consumed Cerifam more than a month ago? Mark all mentioned</td><td><table><tr><th></th><th>Child name 1
Yes=1, No=0</th><th>Child name 2
Yes=1, No=0</th></tr><tr><td>Not available</td><td></td><td></td></tr><tr><td>Expensive</td><td></td><td></td></tr><tr><td>Not needed</td><td></td><td></td></tr><tr><td>Homemade food is better</td><td></td><td></td></tr><tr><td>Don't think it is good</td><td></td><td></td></tr><tr><td>Not aware of the product</td><td></td><td></td></tr><tr><td>Other (specify)…</td><td></td><td></td></tr></table></td></tr>
<tr><td>9.14</td><td>How often do you give Cerifam to (child name)?</td><td><table><tr><th></th><th>Child name 1
Yes=1 No=0</th><th>Child name 2
Yes=1 No=0</th></tr><tr><td>Everyday</td><td></td><td></td></tr><tr><td>Every other day</td><td></td><td></td></tr><tr><td>2 or 3 times per week</td><td></td><td></td></tr><tr><td>One time per week</td><td></td><td></td></tr><tr><td>Less than one time per week</td><td></td><td></td></tr><tr><td>Other(specify)</td><td></td><td></td></tr></table></td></tr>
<tr><td>9.15</td><td>What are the main reasons why you give Cerifam to (NAME)? Mark all mentioned</td><td><table><tr><th></th><th>Child name 1
Yes=1, No=0</th><th>Child name 2
Yes=1, No=0</th></tr><tr><td>Affordable</td><td></td><td></td></tr></table></td></tr>
</table>

		<table><tr><td>Available</td><td></td><td></td></tr><tr><td>Preferred by child</td><td></td><td></td></tr><tr><td>Good for child’s health</td><td></td><td></td></tr><tr><td>Good for child’s strength</td><td></td><td></td></tr><tr><td>Advised by health worker</td><td></td><td></td></tr><tr><td>Advised by family/friends/neighbours</td><td></td><td></td></tr><tr><td>Easy to prepare</td><td></td><td></td></tr><tr><td>Has vitamins and/or minerals</td><td></td><td></td></tr><tr><td>No time to prepare porridge flour at home</td><td></td><td></td></tr><tr><td>Promotion/advertised</td><td></td><td></td></tr><tr><td>Other (specify)</td><td></td><td></td></tr></table>
9.16	What are the reasons why (NAME) has never consumed Cerifam? Mark all mentioned	<table><tr><td></td><td>**Child name 1**<br>Yes=1, No=0</td><td>**Child name 2**<br>Yes=1, No=0</td></tr><tr><td>Not available</td><td></td><td></td></tr><tr><td>Expensive</td><td></td><td></td></tr><tr><td>Not needed</td><td></td><td></td></tr><tr><td>Homemade food is better</td><td></td><td></td></tr><tr><td>Don’t think it is good</td><td></td><td></td></tr><tr><td>Not aware of the product</td><td></td><td></td></tr><tr><td>Other (specify)…</td><td></td><td></td></tr></table>
9.17	The last time that you bought Cerifam, where did you buy it?	Community shop/kiosks…1<br>Retail market…2<br>Wholesale market…3<br>Mini/Supermarkets…4<br>Street vendors…5<br>Door to door trader…6<br>Open market ……..7<br>Other (specify)…8
9.18	The last time that you bought Cerifam, what type of package did you buy?	SHOW OPTIONS<br>200 g sachet…1<br>200 g box…2<br>300 g sachet…3<br>300 g box…4<br>Don’t know/don’t remember…5

9.19	The last time that you bought Cerifam, how much did you pay for it?	______ETB
9.20	Do you give (childnames) other packaged foods similar to Cerifam or Faffa?	**Child name** / **Yes** / **No**<br>Child name 1 / 1 / 0 →skip Q.9.23<br>Child name 2 / 1 / 0 →skip Q..9.23
9.21	Which brand do you buy?	Specify…
9.22	What are the reasons why you buy this/those brand/s as opposed to Cerifam or Faffa?	More affordable…1<br>More available…2<br>Preferred by child…3<br>Better quality…4<br>Advised by health worker…5<br>Promotion/advertised …………..6<br>Other (specify)…7
9.23	Cerifam is planning to distribute a 50 g package just like this one. If this package becomes available, would you be interested in purchasing it?	Yes…1<br>No…2<br>Don't know…3
9.24	How much would you be willing to pay for a 50 g package of Cerifam?	_______ ETB
9.25	Would you be willing to pay 12 ETB for a 50 g package of Cerifam?	Yes…1<br>No…2<br>Don't know…3
9.26	Where would you like to purchase it from	Community shop/kiosks…1<br>Retail market…2<br>Wholesale market…3<br>Mini/Supermarkets…4<br>Street vendors…5<br>Indoor trader…6<br>Other (specify)…7

MODULE 10: CHICKEN/BEEF LIVER, KALE, LENTILS & EGG CONSUMPTION

<table>
<tr><td>10.1</td><td>In the last 7 days…Did you feed eggs to ([childsname1&/or 2])?</td><td>
<table>
<tr><td>Child name</td><td>Yes</td><td>No</td><td>IDK</td></tr>
<tr><td>Child name 1</td><td>1</td><td>0 →skip Q.10.4</td><td>98 →skip Q.10.4</td></tr>
<tr><td>Child name 2</td><td>1</td><td>0 →skip Q.10.4</td><td>98 →skip Q.10.4</td></tr>
</table></td></tr>
<tr><td>10.2</td><td>In the last 7 days…How many times did you feed eggs to ([childsname1&/or 2])?</td><td>
<table>
<tr><td>Child name 1</td><td>______times</td></tr>
<tr><td>Child name 2</td><td>______times</td></tr>
</table></td></tr>
<tr><td>10.3</td><td>In the last 7 days… how many eggs did you feed to ([childsname1&/or 2]) during each time?</td><td>
<table>
<tr><td>Child name 1</td><td>T1_____eggs
T2_____ eggs
T3______ eggs
T4______ eggs
T5______ eggs
T6_____ eggs</td></tr>
<tr><td>Child name 2</td><td>T1_____eggs
T2_____ eggs
T3______ eggs
T4______ eggs
T5______ eggs
T6_____ eggs</td></tr>
</table></td></tr>
<tr><td>10.4</td><td>You mentioned that you did not feed eggs to ([childsname1&/or 2]). What was the reason?</td><td>
<table>
<tr><td></td><td>Child name 1
Yes=1 No=0</td><td>Child name 2
Yes=1 No=0</td></tr>
<tr><td>Not enough money</td><td></td><td></td></tr>
<tr><td>My child doesn’t like egg.</td><td></td><td></td></tr>
<tr><td>I don’t think I should feed egg to my child.</td><td></td><td></td></tr>
<tr><td>My child is too young to eat egg.</td><td></td><td></td></tr>
<tr><td>We don't eat egg in this household</td><td></td><td></td></tr>
<tr><td>I was not able to find egg in the market</td><td></td><td></td></tr>
<tr><td>Egg is too expensive to buy</td><td></td><td></td></tr>
</table></td></tr>
</table>

		Not enough money | | My child doesn't like egg. | | I don't think I should feed egg to my child. | | Other (specify) | |
10.5	How old were your **([childsname1&/or 2])** when you first started feeding them eggs?	**Child name** | **Record in months** | **Never fed** | **IDK** Child name 1 | ______months | 0 | 98 Child name 2 | ______months | 0 | 98
10.6	What type of eggs do you prefer feeding your child?	1 Local eggs 2 Commercial or hybrid eggs 3 Don't have a preference -> **skip to Q.10.8**
10.7	What are the main reasons why you prefer feeding this type of eggs to your child? (mark all mentioned)	It has better taste…1 It has better quality…2 It is bigger…3 It has more vitamins…4 It is easier to find…5 It is cheaper…6 Other (specify)…7
10.8	In what ways are eggs usually prepared in this household?	1 | Hard boiled 2 | Fried 3 | Scrambled 4 | Soft boiled 5 | Mixed with other foods (soups, porridge, noodles, etc) 96 | Other (specify)

10.9	On a scale from 1 to 5, **1** being **"not at all important"** and **5** being **"very important",** how important are the factors in determining whether or not you would purchase eggs for your young child aged 6-59 months?	(see table below)
10.10	Are eggs prepared in a different way for children?	1 Yes<br>0 No
10.11	In what ways are usually prepared for children?	1 Hard boiled<br>2 Fried<br>3 Scrambled<br>4 Soft boiled<br>5 Mixed with other foods (soups, porridge, noodles, etc)<br>96 Other (specify)
10.12	Is there one or more household member who eat eggs more often than others?	1 Yes<br>0 No →**skip module 11**
10.13	Who eats eggs more often?	Father ..1<br>Mother …2<br>Child (6 -59 months) male…3<br>Child (6 -59 months) female…4<br>Above 5 years old children…5<br>Adult male …6<br>Adult female …….7<br>Other
10.14	What is the main reason **[eatname Q10.13]** eats more eggs?	1 Preference<br>2 Health benefits<br>3 Eats outside<br>96 Other (specify)

Q10.9:

	**1**	**2**	**3**	**4**	**5**
Price					
Location of vendors					
Ease of preparation					
Health benefits					
Freshness					
Taste					
Child's preference					
Concerns about egg allergy					
Type of egg (local vs hybrid)					

Module 11: MOTHERS/CAREGIVERS ATTITUDES TOWARDS EGGS (TO BE ASKED OF FEMALE CAREGIVERS)						
On a scale from 1 to 5, 1 being "strongly disagree (SD)" and 5 being "strongly agree (SA)", please tell us how much you agree with each of the following statements.		Strongly disagree	Disagree	Neutral	Agree	Strongly agree
		1	2	3	4	5
11.1	**Attitudes and beliefs**					
	a) Eggs can cause stomach problems among young children					
	b) Children like eggs.					
	c) Young children can choke if they eat eggs.					
	d) Local eggs are natural and healthy.					
	e) Commercial eggs are not natural and should be avoided.					
	f) Eggs can cause problems with digestion and diarrhea.					
	g) Eggs are too "heavy" for infants' stomachs.					
	h) Eggs are a simple meal solution.					
	i) Eggs taste great.					
	j) Eggs are versatile, you can cook them any way you like.					
	k) Eggs are good value for the money.					
	l) Eggs are economical way to add protein to a meal.					
	m) Eggs can cause allergies.					
	n) Children like eggs.					
	o) Eggs are not filling enough.					
	p) Eggs are a luxury item/treat.					
	q) Eggs are for eating outside the home.					
	r) Eggs are for the rich.					
	s) Eggs are too expensive to feed young children.					
	t) Eggs can be mixed with other food items such fruits and vegetables for feeding young children.					
11.2	**Perceived benefits of eggs**					
	a) Eggs are good for brain development of children.					
	b) Eggs strengthen body defense to protect against diseases.					
	c) Eggs make children active and strong.					

	d) Eggs make children sharp and bright.					
**11.3**	**Norms**					
	a) Most children 6-12 months of age in my village eat eggs.					
	b) Most children 1-5 years of age in my village eat eggs.					
	c) I think most of my friends in this community would approve of mothers giving young children egg.					
	d) I believe that my husband/partner would approve of me feeding my children egg.					
	e) I believe that my mother/mother-in-law would approve of me feeding my children egg.					
	f) I believe that my husband/partner/or family will not allow me to cook egg during the fasting season as it will spoil the fast					
**11.4**	**Self-efficacy/Perceived behavioral control**					
	a) I am confident that I can feed egg to my young child.					
	b) It is easy for me to feed egg to my young child.					
	c) The decision to feed egg to my young child is beyond my control.					
	d) Whether I feed egg or not to my young child is not entirely up to me.					
	e) I intend to feed egg to my young child in the next week.					

Module 12: Media Exposure to messages		
12.1	Do you ever watch TV?	Yes…1<br>No …0 → **skip Q.12.3**
12.2	About how many days in a normal week do you watch TV?	_____ days
12.3	Do you ever attend any village gathering (ekub,edier etc)?	Yes…1<br>No …0 → **skip Q.12.5**
12.4	How often do you go to these gatherings?	2 or more times per week…1<br>Once a week….2<br>Once every two weeks….3<br>Once a month ….4<br>Rarely ….5
12.5	Do you ever use social media such as Facebook?	Yes…1<br>No …0 → **skip Q.12.7**
12.6	How often do you use social media?	2 or more times per day….1<br>Once per day….2<br>2 or more times per week….3<br>Once a week….4<br>Once every two weeks….5<br>Once a month….6<br>Rarely….7
12.7	Do you ever listen to the radio?	Yes…1<br>No …0 → **skip Q.12.15**
12.8	About how many days in a normal week do you usually listen to the radio?	____ days
12.9	What radio stations do you listen to the most? (Write the two most important ones)	1…...FM 97.1<br>2… Fana FM 98.1<br>3… Ethio FM<br>4…. Shashemene FM<br>5…. Oromia Radio<br>6…. Mekelle FM<br>7…. Demste Wyane<br>8…. Debub FM<br>98. Other specify______________
12.10	What kind of radio programs do you usually listen to?	News…1<br>Music….2<br>Children’s program…3

		Religious program…4<br>Sports….5<br>Drama….6<br>Health/disease programs….7<br>Other. Specify….98<br>Don't know …..88
12.11	Generally, when do you listen to the radio? (Choose all that apply)	0-5:59 h<br>6:00-11:59 h<br>12:00-17:59 h<br>18:00-23:59 h
12.12	During the past 30 days, did you hear any advertisements about eggs on the radio?	Yes…1<br>No …0 → **skip Q.12.15**
12.13	Do you remember what you heard about egg on the radio?	Yes…1<br>No …0 → **skip Q.12.15**
12.14	What specific message do you remember from what you heard on radio about egg?	Eggs help kids be strong and active<br>Eggs make up a golden lunch<br>Feed eggs multiple times per week<br>Feed eggs to children starting at six months of age<br>Feed eggs with various foods and vegetables<br>Eggs are good for child health<br>Eggs can be produced at home<br>Other (Specify)<br>Don't know/Don't remember
12.15	In the past 30 days, have you received, heard or seen any information about eggs from any sources other than radio?	Yes…1<br>No …0 → **skip Q.12.19**
12.16	From which other sources or persons did you receive information about eggs in the past 30 days?<br>(Multiple response)	(see sub-table below)

1	News Papers		
2	Magazine		
3	Posters at health centres		
4	Posters; egg branded bag, face mask , T-Shirt or cape; umbrellas and/or other materials in markets		
5	Live shows in markets		
6	Household visits		
7	Wall Paintings		

		8	Mobile Phone/SMS		
		9	Cinema		
		10	Leaflet/Brochure		
		11	Health workers		
		12	Community leaders		
		13	Friends		
		14	Neighbours		
		15	Women's Group		
		16	Religious groups		
		17	Children events		
		18	Branded Bajaj		
		19	Community workshops		
		20	Branded vehicle with audio message		
		21	Billboards		
		22	At church or mosque		
		96	Other (Specify)		
		98	Don't know where heard		

12.17	For each of the sources specified above, ask…<br>Do you remember what you heard about egg?	Sources	Yes	No
		News Papers	1	0 → Q. 12.19
		Magazine	1	0 → Q. 12.19
		Posters at health centres	1	0 → Q. 12.19
		Posters; egg branded bag, face mask, T-Shirt or cape; umbrellas and/or other materials in markets	1	0 → Q. 12.19
		Live shows in markets	1	0 → Q. 12.19
		Household visits	1	0 → Q. 12.19
		Wall Paintings	1	0 → Q. 12.19
		Mobile Phone/SMS	1	0 → Q. 12.19
		Cinema	1	0 → Q. 12.19
		Leaflet/Brochure	1	0 → Q. 12.19

		Health workers	1	0 → Q. 12.19
		Community leaders	1	0 → Q. 12.19
		Friends	1	0 → Q. 12.19
		Neighbors	1	0 → Q. 12.19
		Women's Group	1	0 → Q. 12.19
		Religious groups	1	0 → Q. 12.19
		Children events	1	0 → Q. 12.19
		Branded Bajaj	1	0 → Q. 12.19
		Community workshops	1	0 → Q. 12.19
		Branded vehicle with audio message	1	0 → Q. 12.19
		Billboards	1	0 → Q. 12.19
		At church or mosque	1	0 → Q. 12.19
		Other (Specify)	1	0 → Q. 12.19
		Don't know where heard	1	0 → Q. 12.19
12.18	What did you remember about egg?<br>(Multiple response possible)	Eggs help kids be strong and active	1	0
		Eggs make up a golden lunch	1	0
		Feed eggs multiple times per week	1	0
		Feed eggs to children starting at six months of age	1	0
		Feed eggs with other various foods and vegetables	1	0
		Eggs are good for child health	1	0
		Eggs can be produced at home	1	0
		Other (Specify)		
		Don't know /Don't Remember		
12.19	Do you own your own mobile phone?	Yes…1<br>No …0 → **skip Q.12. 23**		
12.20	Is it a basic mobile phone or a smart phone?	1	Basic mobile phone	
		2	Smart phone	
12.21	Do you ever receive SMS messages with advertisement?	Yes…1<br>No …0 → **skip Q.12. 23**		

12.22	How often do you receive SMS messages with advertisement?	2 or more times per day…1<br>Once per day…2<br>2 or more times per week….3<br>Once a week….4<br>Once every two weeks….4<br>Once a month…..5<br>Rarely….6
12.23	Now I would like to ask you whether you have heard any of the following things about feeding your child in the last 30 DAYS:<br><br>(Multiple response)	1 For the first 6 months don't give water to baby<br>2 For the first 6 months give only breastmilk to baby<br>3 Eggs make kids strong and active<br>4 Add fish, meat and vegetable to children's food<br>5 Combine different foods with every meal<br>6 Eggs make kids sharp and bright<br>7 For the first 6 months breastmilk has everything baby needs to grow strong and sharp<br>8 Cook child's food with oil<br>9 Feed child an extra meal or extra food after illness<br>10 Feeding Fafa or cerifam for children is good<br>11 Feed eggs to children starting at six months of age<br>12 Feed eggs with various foods and vegetables<br>13 Feed eggs multiple times per week<br>14 Eggs make up a golden lunch<br>15 Eggs can be produced at home<br>99 Nothing (Not Applicable)
12.24	From whom did you hear about **[exp_12.23]** (*up to 3 options possible*)	

**Message heard**	**Sources**									
	**TV**	**Radio**	**Newspaper/ poster/ billboard**	**Health workers**	**Midwife/ nurse**	**Husband**	**Mother/ mother-in-law**	**School teacher**	**Religious leader**	**Other specify**
For the first 6 months don't give water to baby										
For the first 6 months give only breastmilk to baby										

	Eggs make kids strong and active										
	Add fish, meat, and vegetable to children’s food										
	Combine different foods with every meal										
	Eggs make kids sharp and bright										
	For the first 6 months breastmilk has everything baby needs to grow strong and sharp										
	Cook child’s food with oil										
	Feed child an extra meal or extra food after illness										
	Feeding Fafa or cerifam for children is good										
	Feed eggs to children starting at six months of age										
	Feed eggs with various foods and vegetables										
	Feed eggs multiple times per week										
	Eggs make up a golden lunch										
	Eggs can be produced at home										

12.25

1	News Papers		
2	Magazine		
3	Posters at health centres		
4	Posters, umbrellas and/or other materials in markets		
5	Live shows in markets		
6	Household visits		
7	Wall Paintings		
8	Mobile Phone/SMS		
9	Cinema		
10	Leaflet/Brochure		
11	Health workers		
12	Community leaders		

	From which sources or persons did you receive information about eggs in the past 3 months?<br>(Multiple response)	13	Friends			
		14	Neighbours			
		15	Women's Group			
		16	Religious groups			
		17	Children events			
		18	Branded Bajaj			
		19	Community workshops			
		20	Branded vehicle with audio message			
		21	Billboards			
		22	At church or mosque			
		23	Radio			
		24	Branded bags, bibs, masks, t-shirt, cape			
		25	Facebook			
		96	Other (Specify)			
		98	Don't know where heard			

MODULE 13: HOUSEHOLD FOOD INSECURITY ACCESS		
13.1	In the past four weeks, did you worry that your household would not have enough food?	1 Yes<br>0 No → **Skip Q. 13.3**
13.2	How often does this happen?	1 RARELY (once or twice in the past four weeks)<br>2 SOMETIMES (three to ten times in the past four weeks)<br>3 OFTEN (more than ten times in the past four weeks)
13.3	In the past four weeks, were you or any household member not able to eat the kinds of foods you preferred because of a lack of resources	1 Yes<br>0 No → **Skip Q. 13.5**
13.4	How often does this happen?	1 RARELY (once or twice in the past four weeks)<br>2 SOMETIMES (three to ten times in the past four weeks)<br>3 OFTEN (more than ten times in the past four weeks)
13.5	In the past four weeks, did you or any household member have to eat a limited variety of foods due to a lack of resources?	1 Yes<br>0 No → **Skip Q. 13.7**
13.6	How often does this happen?	1 RARELY (once or twice in the past four weeks)<br>2 SOMETIMES (three to ten times in the past four weeks)<br>3 OFTEN (more than ten times in the past four weeks)
13.7	In the past four weeks, did you or any household member have to eat some foods that you really did not want to eat because of a lack of resources to obtain other types of food?	1 Yes<br>0 No → **Skip Q. 13.9**
13.8	How often does this happen?	1 RARELY (once or twice in the past four weeks)<br>2 SOMETIMES (three to ten times in the past four weeks)<br>3 OFTEN (more than ten times in the past four weeks)
13.9	In the past four weeks, did you or any household member have to eat a smaller meal than you felt you needed because there was not enough food?	1 Yes<br>0 No → **Skip Q. 13.11**
13.10	How often does this happen?	1 RARELY (once or twice in the past four weeks)<br>2 SOMETIMES (three to ten times in the past four weeks)<br>3 OFTEN (more than ten times in the past four weeks)
13.11	In the past four weeks, did you or any other household member have to eat fewer meals in a day because there was not enough food?	1 Yes<br>0 No → **Skip Q. 13.13**

13.12	How often does this happen?	1 RARELY (once or twice in the past four weeks)<br>2 SOMETIMES (three to ten times in the past four weeks)<br>3 OFTEN (more than ten times in the past four weeks)
13.13	In the past four weeks, was there ever no food to eat of any kind in your household because of lack of resources to get food?	1 Yes<br>0 No → **Skip Q. 13.15**
13.14	How often does this happen?	1 RARELY (once or twice in the past four weeks)<br>2 SOMETIMES (three to ten times in the past four weeks)<br>3 OFTEN (more than ten times in the past four weeks)
13.15	In the past four weeks, did you or any household member go to sleep at night hungry because there was not enough food?	1 Yes<br>0 No → **Skip Q. 13.17**
13.16	How often does this happen?	1 RARELY (once or twice in the past four weeks)<br>2 SOMETIMES (three to ten times in the past four weeks)<br>3 OFTEN (more than ten times in the past four weeks)
13.17	In the past four weeks, did you worry that your household would not have enough food?	1 Yes<br>0 No → **Take GPS & End**
13.18	How often does this happen?	1 RARELY (once or twice in the past four weeks)<br>2 SOMETIMES (three to ten times in the past four weeks)<br>3 OFTEN (more than ten times in the past four weeks)
